# 1,1-Bis(3'-indolyl)-1-(*p*-substituted phenyl)methanes induce autophagic cell death in estrogen receptor negative breast cancer

**DOI:** 10.1186/1471-2407-10-669

**Published:** 2010-12-03

**Authors:** Kathy Vanderlaag, Yunpeng Su, Arthur E Frankel, Robert C Burghardt, Rola Barhoumi, Gayathri Chadalapaka, Indira Jutooru, Stephen Safe

**Affiliations:** 1 Department of Veterinary Physiology & Pharmacology, Texas A&M University, College Station, TX 77843-4466, USA; 2 Cancer Research Institute, Scott and White Memorial Hospital, Temple, TX 76502, USA; 3 Department of Veterinary Integrative Biosciences, Texas A&M University, College Station, TX 77843-4458, USA; 4 Institute of Biosciences and Technology, Texas A&M Health Science Center, Houston, TX 77030, USA

## Abstract

**Background:**

A novel series of methylene-substituted DIMs (C-DIMs), namely 1,1-bis(3'-indolyl)-1-(p-substituted phenyl)methanes containing t-butyl (DIM-C-pPhtBu) and phenyl (DIM-C-pPhC6H5) groups inhibit proliferation of invasive estrogen receptor-negative MDA-MB-231 and MDA-MB-453 human breast cancer cell lines with IC50 values between 1-5 uM. The main purpose of this study was to investigate the pathways of C-DIM-induced cell death.

**Methods:**

The effects of the C-DIMs on apoptotic, necrotic and autophagic cell death were determined using caspase inhibitors, measurement of lactate dehydrogenase release, and several markers of autophagy including Beclin and light chain associated protein 3 expression (LC3).

**Results:**

The C-DIM compounds did not induce apoptosis and only DIM-C-pPhCF_3 _exhibited necrotic effects. However, treatment of MDA-MB-231 and MDA-MB-453 cells with C-DIMs resulted in accumulation of LC3-II compared to LC3-I protein, a characteristic marker of autophagy, and transient transfection of green fluorescent protein-LC3 also revealed that treatment with C-DIMs induced a redistribution of LC3 to autophagosomes after C-DIM treatment. In addition, the autofluorescent drug monodansylcadaverine (MDC), a specific autophagolysosome marker, accumulated in vacuoles after C-DIM treatment, and western blot analysis of lysates from cells treated with C-DIMs showed that the Beclin 1/Bcl-2 protein ratio increased.

**Conclusion:**

The results suggest that C-DIM compounds may represent a new mechanism-based agent for treating drug-resistant ER-negative breast tumors through induction of autophagy.

## Background

Studies in this laboratory have investigated the mechanisms of cell death induced by a new series of anticancer agents that are derivatives of phytochemicals expressed in crucifers. Indole-3-carbinol is a phytochemical found as a conjugate in cruciferous vegetables, and both indole-3-carbinol and one of its major metabolites, 3,3'-diindolylmethane (DIM), exhibit a broad range of anticancer and antitumorigenic activities against multiple tumor types [1-6]. Epidemiology studies have correlated consumption of cruciferous vegetables with decreased risk for certain types of cancer [7-11], and indole-3-carbinol and DIM may contribute to cancer chemoprevention associated with these vegetables. The mechanisms of growth inhibition induced by DIM have been well-studied and include G_0_/G_1 _cell cycle arrest, induction of ER stress, induction of apoptosis, activation of aryl hydrocarbon receptor (AhR)-dependent antiestrogenicity, and downregulation of the androgen receptor (AR) [2,5,12-18]. We also synthesized several DIMs substituted in the indole ring and at the methylene carbon bridge to determine structure-activity relationships.

A novel series of methylene-substituted DIMs (C-DIMs), namely 1,1-bis(3'-indolyl)-1-(*p*-substituted phenyl)methanes containing *t*-butyl (DIM-C-pPhtBu) and phenyl (DIM-C-pPhC_6_H_5_) groups, activate peroxisome proliferator-activated receptor γ (PPARγ) and induce receptor-dependent and -independent growth inhibitory and pro-apoptotic responses/genes in colon, pancreatic, ovarian, prostate, bladder and breast cancer cells and/or tumors [19-25]. In ER-negative breast cancer cells, the effect of PPARγ-active C-DIMs on the cell cycle, induction of the pro-apoptotic protein NAG-1, and activation of kinases is primarily receptor-independent and effects of C-DIMs on % distribution of MDA-MB-231 and MDA-MB-453 cells in G_0_/G_1_, S and G_2_/M were minimal [26]. Although C-DIMs modulate Bax and Bcl-2 protein expression, PARP is not cleaved, suggesting a caspase-independent form of cell death [26]. Therefore, the mechanism of cell death induced by C-DIMs in breast cancer cells requires further examination.

In the present study, treatment of ER-negative MDA-MB-231 and MDA-MB-453 cells with C-DIMs did not activate caspases or increase Annexin V staining, indicating that apoptotic cell death was not activated [26]. These observations prompted us to examine other cell death pathways including necrosis and autophagy. The latter pathway is important for cellular homeostasis but can also be activated by some anticancer agents. Measurement of LDH release and propidium iodide (PI) staining suggested that necrosis was not the major form of cell death induced in ER-negative breast cancer cells treated with C-DIMs. In contrast, autophagolysosomes were positively stained with monodansylcadaverine (MDC) after treatment with C-DIMs, and there was a significant increase in LC3b and Beclin 1/Bcl-2 protein ratios. In addition, after treatment with C-DIMs, transfected GFP-LC3 localized to autophagosomal membranes of cells. These data support a contributing role of autophagy in the mechanism of action of C-DIMs in ER-negative breast cancer cells.

## Methods

### Cells, chemicals and other materials

NADH, zVAD-fmk and PI were obtained from Sigma Chemical Co. (St. Louis, MO). MDC was purchased from Fluka (Buchs, Switzerland). The human breast cancer cell lines MDA-MB-231 and MDA-MB-453 were obtained from American Type Culture Collection (Manassas, VA). MDA-MB-231 cells were maintained in DMEM:F-12 supplemented with 0.22% sodium bicarbonate, 10% fetal bovine serum (FBS), and 2 ml/L antibiotic solution (Sigma Chemical Co., St. Louis, MO). MDA-MB-453 cells were maintained in RPMI supplemented with 0.22% sodium bicarbonate, 10% FBS, and 2 ml/L antibiotic solution (Sigma Chemical Co., St. Louis, MO). Cells were grown in 150 cm^2 ^culture plates in an air/CO_2 _(95:5) atmosphere at 37°C and passaged every 5 days. Beclin 1 (H-300) and Bcl-2 (N-19) antibodies were purchased from Santa Cruz Biotechnology (Santa Cruz, CA). The LC3 antibody was purchased from MBL International (Woburm, MA). Horseradish peroxidase substrate for Western blot analysis was purchased from NEN Life Science Products (Boston, MA).

### Cell proliferation assay

MDA-MB-231 and MDA-MB-453 cells were seeded at a density of 3-5 × 10^4^/well in 12-well plates and media was replaced the next day with DMEM:F-12 media containing 2.5% charcoal-stripped FBS and pre-treated with 20 μM zVAD-fmk or vehicle control for 30 min. Cells were then co-treated with zVAD-fmk or vehicle control and DMSO or 10 μM DIM-C-pPhCF_3_, DIM-C-pPhtBu or DIM-C-pPhC_6_H_5 _for 48 h. Cells were then counted using a Coulter Z1 cell counter. Each experiment was completed in triplicate and results are expressed as means ± SE for each determination.

### Annexin V staining

MDA-MB-231 and MDA-MB-453 cells were seeded at a density of 2.5-5 × 10^5^/well in 6-well plates and media was replaced the next day with DMEM:F-12 media containing 2.5% charcoal-stripped FBS and DMSO or 10 mM DIM-C-pPhCF_3_, DIM-C-pPhtBu or DIM-C-pPhC_6_H_5 _for 48 h. Cells were also treated for 24 h with 10 mM MG132. Cells were then harvested according to the Annexin-V-FITC protocol provided by BD Biosciences. Briefly, floating cells were transferred to an Eppendorf tube and centrifuged for 2 min at 3000 *g*. The adherent cells were trypsinized and transferred to the same Eppendorf which was spun and the supernatant was removed. Cells were resuspended in 85 ml of 1× Annexin V binding buffer and 10 ml of PI solution (5 μg/mL). Five ml of Annexin V-FITC conjugate was added to each sample and then incubated in the dark for 15 min before analysis by flow cytometry which may underestimate the number of dead cells due to fragmentation of floating cells. Cells early in apoptosis positively stained for Annexin V, whereas cells in the later stages of apoptosis stained positively for Annexin V and PI due to late apoptotic cells having leaky plasma membranes (magnification = 400×).

### PI and Hoechst staining

Vybrant Apoptosis Assay Kit #7 was used from Molecular Probes (Eugene, OR) according to the manufacturer's directions. Briefly, monolayers of cells were cultured for 48 h in 2-well Coverglass Chamber slides. Slides were washed with culture medium without serum or phenol red, and labeled with Hoechst 33343 and propidium iodide at a final concentration of 5 μg/ml and 1.0 μg/ml, respectively. The slides were incubated for 30 min on ice and visualized using a BioRad Radiance 2000 Multiphoton microscope. At least 3 areas per well were analyzed. Two wells were analyzed per treatment and per time point.

### LDH assay

MDA-MB-231 and MDA-MB-453 cells were treated for 48 h with DMSO or C-DIMs as indicated. An extra 3 wells were treated for 1 h or until all cells had lysed with 0.3% Triton X in PBS. For each sample, 200 μl of 1.22 mM pyruvate in 50 mM phosphate buffer and 4 μl of 12.4 mg/ml NADH dissolved in 50 mM phosphate buffer were each added to a 96-well plate. Twenty μl of supernatant from treated cells was then added and the plate was incubated at 37°C for 30 min. The LDH concentration was measured at 390 nm and the treated groups were compared to Triton X, which was considered 100% LDH release.

### MDC staining

Monolayers of cells were cultured for 48 h in 2-well Coverglass Chamber slides and treated as indicated. Slides were washed with culture medium without serum or phenol red. Cytoplasmic vacuoles were stained with MDC according to the method described [27]. Briefly, cells were exposed to 50 mM of MDC for 10 min at 37°C and visualized using a BioRad Radiance 2000 Multiphoton microscope. At least 3 areas per well were analyzed. Two wells were analyzed per treatment and per time point (magnification = 400×).

### Western blots

MDA-MB-231 and MDA-MB-453 cells were seeded in DMEM:F-12 media containing 2.5% charcoal-stripped FBS for 24 h and then treated with either the vehicle (DMSO) or the indicated compounds. In experiments where indicated, cells were pre-treated for 30 min with 10 mM of the proteasome inhibitor MG132. Whole cell lysates were obtained using high salt buffer [50 mM HEPES, 500 mM NaCl, 1.5 mM MgCl_2_, 1 mM EGTA, 10% glycerol and 1% Triton X-100 (pH 7.5) and 5 μl/ml of Protease Inhibitor Cocktail]. Protein samples were incubated at 100°C for 2 min, separated on 10-15% SDS-PAGE at 120 V for 3-4 h in 1× running buffer [25 mM Tris-base, 192 mM glycine, and 0.1% SDS (pH 8.3)] and transferred to a polyvinylidene difluoride (PVDF) membrane at 0.9 A for 90 min at 4°C in 1× transfer buffer (48 mM Tris-HCl, 39 mM glycine, and 0.025% SDS). The PVDF membrane was blocked in 5% TBST-Blotto [10 mM Tris-HCl, 150 mM NaCl (pH 8.0), and 0.025% Triton X-100 and 5% non-fat dry milk] with gentle shaking for 30 min and incubated in fresh 5% TBST-Blotto with 1:200-1:1000 primary antibody overnight at 4°C with gentle shaking. After washing with TBST for 10 min, the PVDF membrane was incubated with secondary antibody (1:5000) in 5%TBST-Blotto for 2 h. The membrane was washed with TBST for 10 min and incubated with 10 ml of chemiluminescence substrate for 1.0 min and exposed to Kodak X-OMAT AR autoradiography film. Band intensities were evaluated by scanning laser densitometry (Sharp Electronic Corporation, Mahwah, NJ) using Zero-D Scanalytics software (Scanalytics Corporation, Billerica, MA).

### GFP-LC3 localization

Monolayers of cells were cultured for 48 h in 2-well Coverglass Chamber slides and treated as indicated. The GFP-LC3 plasmid was kindly provided by Dr. Tamotsu Yoshimori (Osaka University, Osaka, Japan). MDA-MB-231 cells were transfected with 500-600 ng/well of GFP-LC3 plasmid using Lipofectamine transfection reagent (Invitrogen, Carlsbad, CA) according to the manufacturer's protocol. MDA-MB-453 cells were transfected with 600 ng/well of GFP-LC3 plasmid using GeneJuice transfection reagent (EMD Biosciences, Madison, WI) according to the manufacturer's protocol. Cells with GFP-LC3 expression were counterstained with Hoechst DNA dye from Molecular Probes (Eugene, OR). Slides were examined by fluorescence microscopy using a Zeiss Stallion Dual Detector Imaging System (Carl Zeiss Microimaging Inc., Thornwood, NY). The intracellular distribution of GFP-LC3 was evaluated by monitoring GFP-LC3 and Hoechst fluorescence and DIC images throughout the entire thickness of the cell by acquiring optical slices at 0.5 μm intervals using a C-Apochromat 63×, 1.2 NA water immersion lens. Digital images were acquired using Slide Book software (Intelligent Imaging Innovations, Denver, CO). The entire z-stack was subjected to fluorescence deconvolution to remove out-of-plane fluorescence. Cells were examined in more than 5 fields per slide on multiple slides. Data represents the average of all the fields.

### Beclin-1 immunohistochemistry in tumors

The generation of tumors was performed as described previously [26] in 4-6 week old female Balb/c athymic nude mice (Nu-/-). All procedures were performed in accordance with the National Institutes of Health "Guide for the Care and Use of Laboratory Animals" and were approved by the Institutional Animal Care and Use Committee of Texas A&M University. Briefly, mice were injected subcutaneously in the left flank with 10^7 ^MDA-MB-231 cells in 100 - 200 μl serum-free medium. Groups of mice (12 mice/group) were then treated i.p. with 40 mg/kg DIM-C-pPhC_6_H_5 _in 50 μl corn oil, or 50 μl corn oil, every day for 35 total injections starting at day 4 post-tumor inoculations.

Tumor Beclin-1 expression was evaluated on 7-μm thick formalin-fixed, paraffin-embedded tissue sections by a standard immunohistochemistry protocol. Briefly, endogenous peroxidase was blocked by the use of 3% hydrogen peroxide in PBS for 10 min. The slides were then washed with distilled water for 2 min and this washing step was repeated twice. Slides were then incubated for 30 min at room temperature with a protein blocking solution (VECTASTAIN Elite ABC kit, Vector Laboratories, Burlingame, CA). Excess blocking solution was drained, and the samples were incubated overnight at 4°C at a 1:100 dilution of anti-rabbit Beclin 1 antibody. Sections were then incubated with biotinylated secondary antibody followed by streptavidin (VECTASTAIN Elite ABC kit, Vector Laboratories, Burlingame, CA). The color was developed by exposing the peroxidase to diaminobenzidine reagent (Vector Laboratories, Burlingame, Ca), and Beclin 1 expression was identified by the brown-colored cytoplasmic staining. Tumors from two different animals per treatment group were evaluated.

### Statistical analysis

Statistical differences between different groups were determined by ANOVA followed by Bonferroni post hoc test for multiple comparisons at P < 0.05 using SuperAnova software. The data are presented as mean ± standard error for at least 3 separate determinations for each treatment.

## Results

**Figure 1:**
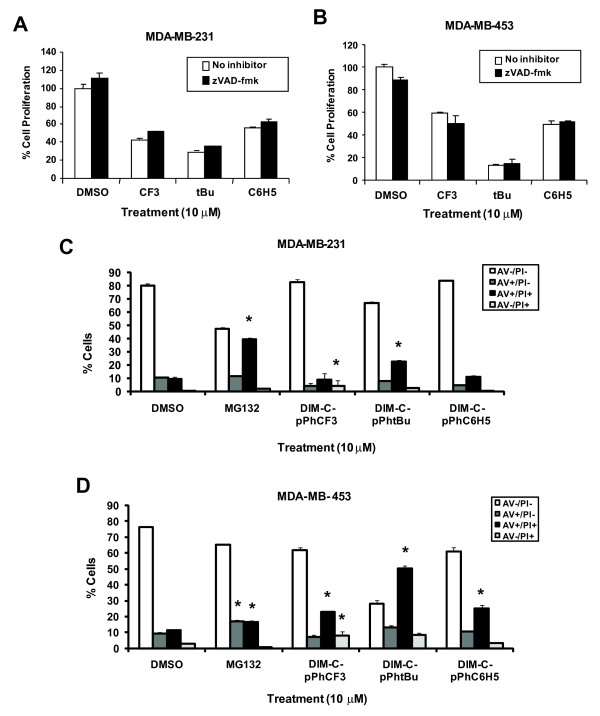
**Influence of pan-caspase inhibitor zVAD-fmk on growth inhibition induced by C-DIMs**. MDA-MB-231 [A] and MDA-MB-453 [B] cells were treated for 48 h with 10 μM DIM-C-pPhCF_3 _(CF_3_), DIM-C-pPhtBu (tBu) or DIM-C-pPhC_6_H_5 _(C_6_H_5_) and with or without 20 μM zVAD-fmk and then counted as described in the Materials and Methods. Results of all proliferation studies are presented as means ± SE for at least three separate determinations for each treatment group. Significantly (p < 0.05) decreased growth (compared to DMSO) in cells treated with C-DIMs alone is indicated (*). FACS analysis of MDA-MB-231 [C] and MDA-MB-453 [D] cells co-stained with Annexin V and PI. Cells were treated for 48 h or 24 h with 10 μM MG132. Floating and adherent cells were incubated with Annexin V and PI and analyzed by flow cytometry as described in the Materials and Methods. Results of all proliferation studies are presented as means ± SE for at least three separate determinations for each treatment group. Significance (p < 0.05) compared to DMSO is indicated (*).

PPARγ-active C-DIMs inhibit growth and activate caspase-dependent apoptosis in colon cancer cells [23]. C-DIMs also inhibit growth of MDA-MB-231 and MDA-MB-453 breast cancer cells; however, this was not accompanied by caspase-dependent PARP cleavage [26]. Figure 1A and 1B show that there was no statistically significant reversal of C-DIM-mediated cell growth inhibition after co-treatment of MDA-MB-231 and MDA-MB-453 cells with the pan-caspase inhibitor zVAD-fmk, suggesting that growth inhibition by the C-DIM compounds was apoptosis-independent.

MDA-MB-231 and MDA-MB-453 cells were treated with C-DIMs for 48 h, co-stained with Annexin V-FITC conjugate which is a marker of apoptosis and PI and analyzed by flow cytometry (Figure 1C and 1D). PI is a DNA dye that is impermeable to cells with intact membranes and is a marker of late apoptotic or necrotic cells that have lost membrane integrity. Early apoptotic cells stain positive for Annexin V, whereas cells in the later stages of apoptosis stain positively for both Annexin V and PI. The majority of cells treated with solvent control DMSO clustered in the quadrant negative for both Annexin V and PI. After treatment with the apoptosis-inducing compound MG132, there was a slight increase in MDA-MB-231 (but not MDA-MB-453) cells staining positively for Annexin V alone and a statistically significant increase in cells staining positive for Annexin V and PI in both cell lines, suggesting that cells treated with MG132 were in the late stages of apoptosis. In MDA-MB-231 cells treated with C-DIMs, there was a decrease in the number of cells staining positively for Annexin V alone, and increased staining for Annexin V and PI was observed only for DIM-C-pPhtBu. In both MDA-MB-231 and MDA-MB-453 cells staining for PI alone was observed only after treatment with DIM-C-pPhCF_3_. In addition, all 3 C-DIM compounds significantly increased Annexin V and PI staining in MDA-MB-453 cells, indicating that these cells exhibited the prototypical characteristics of late stage apoptosis and necrosis (Figure 1D). The failure of C-DIMs to induce Annexin V alone indicates that the ER-negative breast cancer cells do not undergo the early stages of apoptosis and this was consistent with previous studies in these cell lines treated with C-DIMs for only 24 h [26]. The increased number of cells staining positive for both Annexin V and PI are indicative of C-DIM-induced loss of cell membrane integrity but Annexin V/PI staining does not identify which cell death pathways are activated.

**Figure 2:**
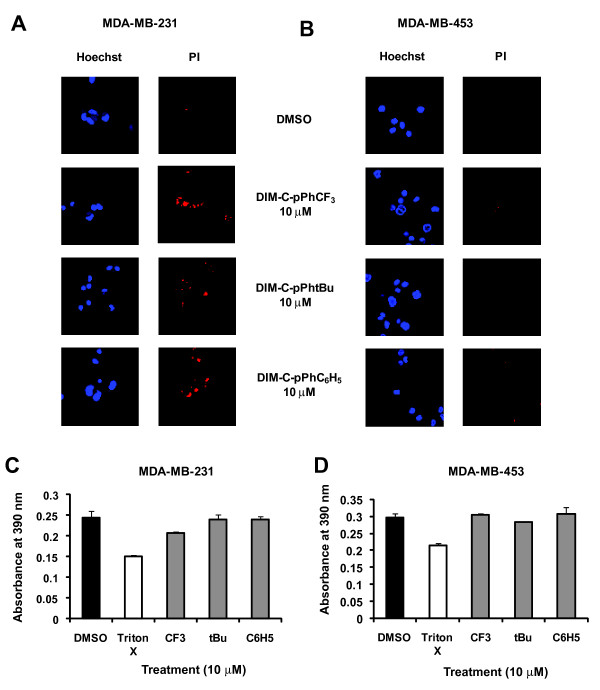
**Necrosis detection by PI staining and LDH release**. MDA-MB-231 [A] and MCF-7 [B] cells were treated for 24 h with DMSO or 10 μM DIM-C-pPhCF_3_, DIM-C-pPhtBu or DIM-C-pPhC_6_H_5_. Two fluorescent DNA dyes Hoechst and propidium iodide, were loaded into cells and incubated on ice for 30 min and visualized using confocal microscopy as described in the Materials and Methods. The same microscopic field of MDA-MB-231 [A] or MDA-MB-453 [B] cells stained with Hoechst and propidium iodide are shown and are representative of other cell areas with the same treatment. At least 3 areas were scanned for each well and two wells were analyzed per treatment and per time point. Width of each field = 100 μm. LDH release after treatment with C-DIMs in MDA-MB-231 [C] and MDA-MB-453 cells [D]. Cells were treated for 48 h or 1 h with 0.1% Triton X and the supernatants were analyzed for LDH as described in the Materials and Methods. Triton X served as a positive for 100% cell lysis and LDH release. Results are presented as the means ± SE for at least three separate determinations for each treatment group. Statistical significance of treatments compared to DMSO (p < 0.05) are represented by an asterisk.

Some MDA-MB-231 cells were stained with PI after treatment with C-DIMs; however, PI staining was not observed in MDA-MB-453 cells treated with C-DIMs (Figure 2A and 2B). LDH release from cells was significantly elevated after treatment with the lytic agent, Triton X in both cell lines; however, among the three PPARγ-active C-DIMs only DIM-C-pPhCF_3 _significantly increased levels of LDH release in MDA-MB-231 cells, whereas none of the PPARγ-active C-DIMs induced LDH release in MDA-MB-453 cells (Figure 2C and 2D). Thus, only DIM-C-pPhCF_3 _significantly induced necrosis in one of the two cell lines, suggesting that necrosis was not the predominant death pathway induced by PPARγ-active C-DIMs in ER-negative breast cancer cells.

**Figure 3:**
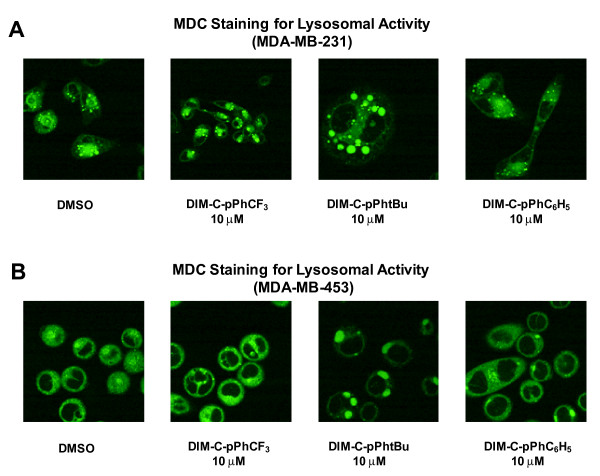
**Detection of lysosomal activity with monodansylcadaverine (MDC) staining**. MDA-MB-231 [A] and MDA-MB-453 cells [B] were treated with DMSO or 10 μM DIM-C-pPhCF_3_, DIM-C-pPhtBu or DIM-C-pPhC_6_H_5 _for 24 h. MDC was loaded into cells and incubated at 37°C for 10 min and visualized using confocal microscopy as described in the Materials and Methods. At least 5 areas were scanned for each well and images represented are characteristic of treatment replicates. Width of each field is 100 μm except for right two panels in [A] which are 50 μm.

Several drugs that are cytotoxic to cancer cell lines induce autophagic cell death [28-38] and, therefore, the effects of C-DIMs on MDC localization, a positive marker for autophagolysosomes, was investigated. The C-DIMs clearly induced MDC localization in vacuoles in MDA-MB-231 cells, whereas in cells treated with DMSO, a smaller number and size of vacuoles was observed indicating low basal levels of autophagy in these cells (Figure 3A). Thus, treatment with C-DIMs increased the number and/or size of vacuoles in this cell line. MDC was also localized in large vacuoles in MDA-MB-453 cells treated with DIM-C-pPhtBu and DIM-C-pPhC_6_H_5 _(Figure 3B), whereas vacuole size and number in cells treated with DIM-C-pPhCF_3 _resembled the control cells. These results suggest that DIM-C-pPhtBu and DIM-C-pPhC_6_H_5 _induced autophagy in MDA-MB-231 and MDA-MB-453 cells.

**Figure 4:**
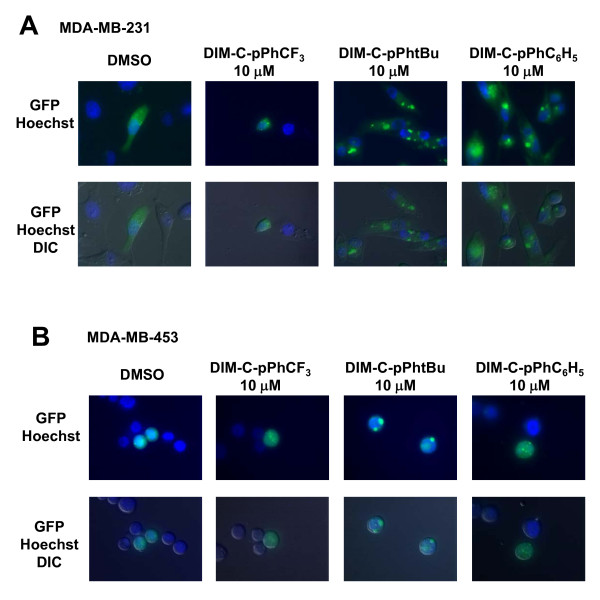
**Translocation of GFP-LC3 after treatment with C-DIMs**. Translocation of transiently transfected GFP-LC3 plasmid. GFP-LC3 was transiently transfected into MDA-MB-231 [A] and MDA-MB-453 [B] and cells were treated with DMSO or 10 μM C-DIMs as outlined in the Materials and Methods. Confocal microscopy images of live cells were captured after treatment with C-DIMs for 24 h and differential interference contrast (DIC) and DIC/GFP overlay images of the same microscopic field are illustrated. At least 3 areas were scanned per well and the images depicted are representative of treatment replicates. Width of each field is 100 μm.

LC3b is critical for autophagosome formation and after induction of autophagy, LC3b-I is lipidated to LC3b-II which localizes to autophagosomal membranes, and the ratio of LC3b-II to LC3b-I protein expression is an indicator of autophagosome formation [39]. Confocal microscopy images of a GFP-LC3 construct transiently transfected into the two cell lines showed that in untreated or DMSO-treated cells, there was a diffuse GFP staining pattern (punctae), whereas after treatment with C-DIMs, GFP-LC3 was primarily localized in vacuoles (Figure 4A and 4B). In MDA-MB-231 cells, there was an increase in the number of vacuoles positively stained for GFP-LC3 after treatment with DIM-C-pPhtBu and DIM-C-pPhC_6_H_5 _compared to DMSO (Figure 4A). The effect of DIM-C-pPhCF_3 _was less pronounced than with the other two compounds in MDA-MB-231 cells and this was consistent with the higher necrotic activity of this compound (Figure 2). In MDA-MB-453 cells, the size of the vacuoles was substantially increased after treatment with DIM-C-pPhtBu, whereas only the number of vacuoles increased in cells after treatment with DIM-C-pPhC_6_H_5 _(Figure 4B). These data suggest that C-DIM compounds activated autophagic cell death pathways in MDA-MB-231 and MDA-MB-453 cells.

**Figure 5:**
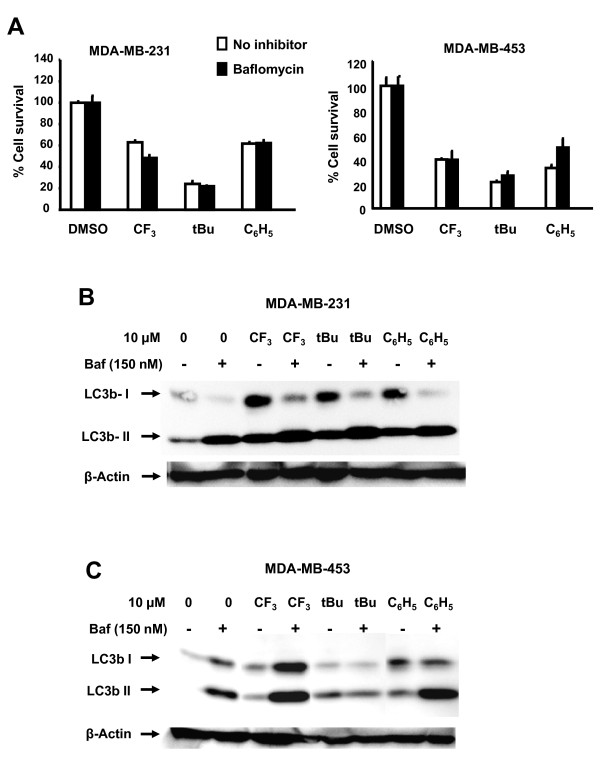
**Effects of bafilomycin on C-DIM-induced responses**. [A] Growth inhibition in breast cancer cells. MDA-MB-231 and MDA-MB-453 cells were treated with DMSO, C-DIMs alone (10 μm), or in combination with bafilomycin (150 nM) for 48 hr, and cells were counted as described in the Materials and Methods. Results are expressed as means ± SD for replicate (3) determinations. Effects of bafilomycin on LC3b protein in MDA-MB-231 [B] and MDA-MB-453 [C] cells. Cells were treated with DMSO, C-DIMs alone, or in combination with bafilomycin for 48 hr, and whole cell lysates were analyzed by western blots as outlined in the Materials and Methods.

Bafilomycin A1 inhibits maturation of autophagosomes into autolysosomes by inhibiting fusion between autophagosomes and lysosomes [40] and this compound was used to further investigate the role of autophagy in C-DIM-induced growth inhibition. Figure 5A shows that DIM-C-pPhCF_3_, DIM-C-pPhtBu and DIM-C-pPhC_6_H_5 _alone inhibit growth of MDA-MB-231 and MDA-MB-453 cells. Their growth inhibitory effects were not affected after treatment in combination with bafilomycin and similar effects were observed with 3-methyladenine (data not shown). These results indicate that induction of autophagy by C-DIMs is not important for their inhibition of cell growth. In untreated MDA-MB-231 cells, both LC3b-I and LC3b-II proteins are expressed; treatment with DIM-C-pPhCF_3_, DIM-C-pPhtBu and DIM-C-pPhC_6_H_5 _increased expression of both proteins. Bafilomycin alone increased accumulation of LC3b-II protein and in combination with the C-DIM compounds, LC3b-II levels were further increased compared to bafilomycin alone, suggesting activation of autophagy by the C-DIMs in this cell line. With the exception of DIM-C-pPhtBu, similar results were observed in MDA-MB-453 cells treated with bafilomycin alone or in combination with C-DIMs. It is possible that DIM-C-pPhtBu may affect clearance of the autophagic vesicles.

**Figure 6:**
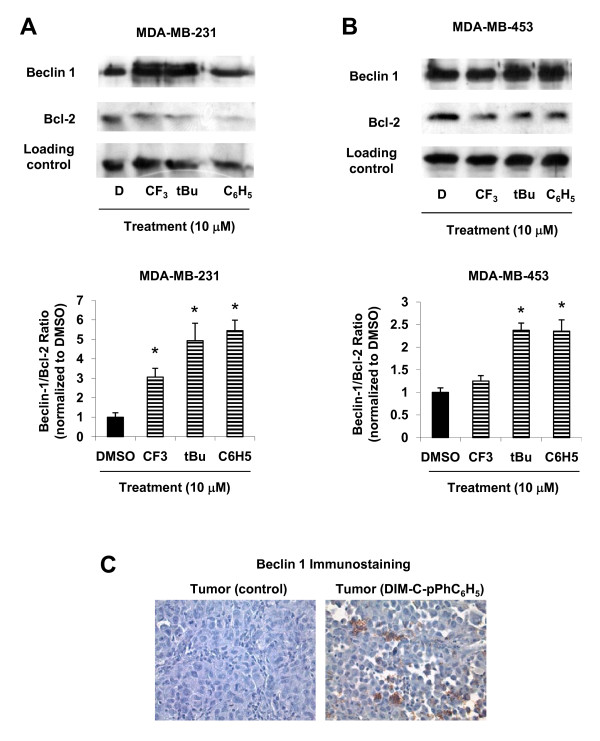
**Modulation of autophagy-related gene Beclin 1 and Bcl-2 protein expression**. MDA-MB-231 [A] and MDA-MB-453 [B] cells were treated for 24 h with DMSO or C-DIMs, and whole cell lysates were isolated and analyzed by Western blot analysis according to procedures outlined in the Materials and Methods. Densitometric analyses of Beclin 1 and Bcl-2 protein expression in MDA-MB-231 and MDA-MB-453 cells was carried as described in the Materials and Methods. Results are expressed as means ± SE for three replicate determinations for each treatment group. * Significantly different from DMSO (p < 0.05). [C] Enhanced tumor staining of Beclin 1 after treatment with DIM-C-pPhC_6_H_5 _in nude mice bearing MDA-MB-231 cells as xenografts. Fixed tumor tissue from corn oil and DIM-C-pPhC_6_H_5_-treated mice [26] were stained with Beclin 1 as described in the Materials and Methods. Results are representative of tumors from two different animals per treatment group. Width of each field is 230 μm.

Although the same autophagy-related genes (Atg5, Atg6, Atg7) play a role in autophagy and autophagic cell death, both Atg5 and Atg6/Beclin 1 are upregulated in autophagic cell death and remain low in autophagy [41]. The anti-apoptotic protein Bcl-2 has an inhibitory action on Beclin 1 and the subsequent induction of autophagy [42] and, therefore, the effects of C-DIMs on expression of these proteins were further investigated. Treatment of MDA-MB-231 cells with C-DIMs increased Beclin 1 and decreased Bcl-2 protein levels (Figure 6A), and in MDA-MB-453 cells treated with C-DIMs, Beclin 1 protein levels increased only after treatment with DIM-C-pPhtBu and DIM-C-pPhC_6_H_5 _and Bcl-2 protein was decreased (Figure 6B). Densitometric analyses of these proteins showed that C-DIMs increased Beclin 1/Bcl-2 ratios in MDA-MB-231 compared to that observed for DMSO (Figure 6A); however, a statistically significant increase in Beclin 1/Bcl-2 protein ratios was observed only in MDA-MB-453 cells treated with DIM-C-pPhtBu and DIM-C-pPhC_6_H_5 _(Figure 6B). *In vivo *studies showed that DIM-C-pPhC_6_H_5 _inhibited growth of tumors in nude mice bearing MDA-MB-231 cells as xenografts [26]. We analyzed induction of autophagy in these tumors after treatment with DIM-C-pPhC_6_H_5 _and the results (Figure 6C) show enhanced staining of Beclin 1 in tumors from treated animals versus mice receiving only corn oil (vehicle control). These results suggest that C-DIMs induce cell death (Figure 1) in ER-negative breast cancer cells and this may be partially due to activation of autophagy and this demonstrates the versatility of this novel class of anticancer drugs which activate multiple receptor-independent cell death pathways in different cancer cell lines.

## Discussion

C-DIMs inhibit ER-negative MDA-MB-231 and MDA-MB-453 breast cancer cell proliferation and tumor growth in athymic nude mice bearing MDA-MB-231 cells as xenografts. C-DIMs represent a novel series of compounds that exert their growth inhibitory and antitumorigenic effects in a cell context-dependent manner. C-DIMs activate PPARγ in a number of cancer cell types including ovarian, bladder, colon, pancreatic and ER-negative and ER-positive breast cancer cells [19-22,24,25]. PPARγ agonists inhibit cancer growth by molecular mechanisms that include G_1 _cell cycle arrest, induction of apoptosis and terminal differentiation [43-46]. PPARγ-active C-DIMs and other PPARγ agonists also activate PPARγ-independent growth inhibitory pathways including induction of NAG-1, ER stress and apoptosis [20,47-52]. C-DIMs induce NAG-1 which is proapoptotic in some cancer cell lines; however, in MDA-MB-231 and MDA-MB-453 cells, these compounds induced NAG-1 but not apoptosis. In addition, induction of other receptor-dependent and -independent pro-apoptotic and differentiation pathways such as ER stress and caveolin 1 was not observed in ER-negative breast cancer cells. Thus, the precise mechanism of C-DIM-induced cell death in ER-negative breast cancer cells is unclear and multiple pathways may be involved.

EB1089 is a vitamin D analog that induces caspase-independent cell death in ER-positive MCF-7 and ER-negative MDA-MB-231 and MDA-MB-453 breast cancer cells, and similar results were observed for C-DIMs which also induced caspase-independent growth inhibition which could not be reversed after co-treatment with the pan-caspase inhibitor zVAD-fmk (Figure 1). We also confirmed that treatment with C-DIMs for 48 h did not induce Annexin V staining indicative of early apoptotic cells in ER-negative breast cancer cells, whereas the pro-apoptotic agent MG132 induced cells staining with Annexin V staining in cells (Figure 1). These results coupled with previous studies showing that treatment with C-DIMs for 24 h did not induce caspase-dependent PARP cleavage in these cell lines [26] confirm that these compounds primarily induce apoptosis-independent cell death. Other chemotherapeutic agents such as the cytotoxic drug paclitaxel and related analogs induced cell death in breast cancer cells; however, only a small percentage of cells were undergoing apoptosis [29]. Thus, the current studies suggest that an alternative form of cell death is induced by C-DIMs in ER-negative breast cancer cells and this contrasts with the induction of caspase-dependent apoptosis in ER-positive MCF-7 cells treated with C-DIMs [25].

Necrosis is an alternative form of cell death and although initially considered an uncontrolled form of cell demise, there is increasing evidence supporting the concept of programmed necrosis [53]. For instance, DNA damaging agents induced programmed cell death in Bax^-/-^Bak^-/- ^cells but only in actively proliferating cells and these observations suggested an intrinsic cellular control point that decides cellular fate [54]. This form of cell death is particularly appealing for chemotherapeutic agents since many tumor cells have dysfunctional apoptotic pathways and, therefore, apoptosis-inducing agents are not always effective for treating cancer [53]. In MDA-MB-231 cells, minimal staining of PI and a statistically significant release of LDH was observed only after treatment with DIM-C-pPhCF_3_; signs of necrosis (PI staining) were also observed in MDA-MB-231 cells treated with DIM-C-pPhtBu or DIM-C-pPhC_6_H_5 _(Figure 2A and 2C). The lack of LDH release and PI staining in MDA-MB-453 cells treated with C-DIMs (Figure 2B and 2D) suggested that necrosis was not significantly induced in this cell line. These results also indicate some structure-dependent differences between C-DIMs and also with the structurally-related ring-substituted DIMs. For example, 5,5'-dibromoDIM induced necrotic cell death in ER-negative MDA-MB-453 cells, whereas DIM-C-pPhtBu and DIM-C-pPhC_6_H_5 _did not activate this cell death pathway [12].

Autophagic cell death is an alternative form of programmed cell death where cells lack the hallmarks of apoptosis but there is an accumulation of autophagic vacuoles in the cytoplasm [55], and several structurally diverse chemotherapeutic agents induce autophagic cell death in various cancer cell lines [28-30,32-34]. For example, the PPARγ agonist prostaglandin J2 induced autophagic cell death in prostate cancer cells [34] and the phytochemical sulforaphane induced autophagic cell death in prostate cancer cells [28]. Therefore, we also examined the effects of C-DIMs on activation of autophagy in MDA-MB-231 and MDA-MB-453 breast cancer cells. There are several biochemical methods for detecting autophagic activity including acidic dyes that label vacuoles which exhibit lysosomal activity [27]. Autophagic cell death in prostate cancer cells treated with sulforaphane exhibited lysosomotropic staining of cytoplasmic vacuoles with acridine orange [28]. Other chemotherapeutic candidate drugs induce signs of autophagy in breast cancer cells [29,32,37,56]. Prenylated flavones inhibited cell growth and induced autophagy in both ER-positive MCF-7 and ER-negative MDA-MB-231 breast cancer cells and this response was typified by the formation of cytoplasmic vacuoles that stained with the autophagic marker MDC [56]. The vitamin D analog EB1089 also inhibited cell growth in MCF-7 breast cancer cells and increased uptake of MDC into cytoplasmic vacuoles [37]. These observations were similar to effects of C-DIMs on ER-negative breast cancer cells which also exhibited increased uptake of MDC into vacuoles (Figure 3), suggesting that these compounds induce autophagy. Therapeutic agents such as EB1089 and prenylated flavones typically induce punctate lysosomotropic staining, whereas treatment with DIM-C-pPhtBu in particular induced formation of very large MDC-stained vacuoles (Figure 3). These differences in staining may be due, in part, to differences in the size of autophagosomes in various cancer cell lines [57] caused by differences in autophagic flux.

LC3b, a critical protein involved in the early stage of autophagosome formation, becomes lipidated upon induction of autophagy [58]. Induction of LC3-II protein expression, an increase in the ratio of LC3b-II/LC3b-I expression, and translocation of LC3 to autophagosomal membranes are diagnostic molecular markers indicative of autophagy [39]. For example, treatment of breast cancer cells with camptothecin or paclitaxel analogs increase the ratio of LC3b-II/LC3b-I protein expression [29,59]. With the exception of DIM-C-p-PhCF_3 _in MDA-MB-453 cells, treatment with C-DIMs induced translocation of GFP-LC3 from the cytoplasm to autophagosomal membranes (Figure 4A and 4B). Chemotherapeutic agent EB1089 or radiation that induce autophagic cell death typically induce GFP-LC3 staining patterns in breast cancer cells similar to those observed in this study with C-DIMs [33,37]. Bafilomycin blocks maturation of autophagosomes and in combination with C-DIMs did not inhibit or enhance their growth inhibitory effects in MDA-MB-231 and MDA-MB-453 cells (Figure 5A). However, bafilomycin alone increased accumulation of LC3b-II in both cell lines (Figure 5B and 5C) and with the exception of DIM-C-pPhtBu (MDA-MB-453 cells), the C-DIM compounds further enhanced bafilomycin-induced LC3b-II levels, suggesting activation of autophagy in these cells.

An important difference between autophagy as a survival mechanism versus autophagic cell death involves the Atg5 and Atg6/Beclin 1 genes, which are upregulated in autophagic cell death [41]. The anti-apoptotic protein Bcl-2 is also critically involved as a negative regulator of the induction of autophagy by Beclin 1 [42]. Ceramide [60], camptothecin [61], and EB1089 [37] induce autophagic cell death in breast cancer cells and all of these agents increase Beclin 1 protein expression. These observations are similar to the significant increase of Beclin 1 protein levels and Beclin 1/Bcl-2 ratios in MDA-MB-231 and MDA-MB-453 cells after treatment with C-DIMs (Figure 6A and 6B) and also in tumors from mice bearing MDA-MB-231 cells as xenografts and treated with DIM-C-pPhC_6_H_5 _(Figure 6C). While further studies that explore autophagic flux and the actions of other autophagy inhibitors on C-DIM growth inhibition are warranted, these findings represent the first report to document the activation of an autophagic program in ER-negative breast cancer cells with C-DIMs.

## Conclusions

In summary, results of this study demonstrate that C-DIM compounds clearly decrease ER-negative breast cancer cell proliferation and like some other cancer chemotherapeutic drugs induce autophagy. These results are in contrast to the mechanisms of action of C-DIMs in colon, pancreatic, ovarian, prostate and bladder cancer cell lines where these same compounds induce both PPARγ-dependent and -independent growth inhibitory and pro-apoptotic responses [19,20,22-25,47]. The mechanisms associated with the cell context-dependent differences in the anticancer activities of C-DIMs and their mechanisms of growth inhibition and cell death in ER-negative breast cancer cells are currently being further investigated. The unusual activity of C-DIMs is also being exploited for the clinical treatment of ER-negative/highly invasive breast tumors that normally only respond to highly cytotoxic drugs which induce serious toxic side effects.

## Abbreviations

AHR: aryl hydrocarbon receptor; AR: androgen receptor; C-DIMS: methylene-substituted DIMs; DIM: 3,3'-diindolylmethane; DIM-C-PPHC_6_H_5_: 1,1-bis(3'-indolyl)-1-(*p*-phenyl)methane; DIM-C-PPHTBU: 1,1-bis(3'-indolyl)-1-(*p*-*t*-butyl)methane; FBS: fetal bovine serum; MDC: monodansylcadaverine; PI: propidium iodide; PPARγ: peroxisome proliferator-activated receptor γ; PVDF: polyvinylidene difluoride

## Competing interests

The authors reports no conflicts of interest in this work; however, the C-DIM compounds have been licensed from Texas A&M University by Plantacor (College Station, TX).

## Authors' contributions

KV carried out the majority of the *in vitro *studies, analyzed and summarized the results, and drafted the manuscript. YS and AF carried out *in vitro *studies and were involved in the design and implementation of the anticancer activities of C-DIMs. RCB and RB collaborated in all of the imaging studies. GC and IJ carried out the studies with bafilomycin and 3-methyladenine. SS coordinated the study, synthesized the compounds, and helped to draft the manuscript. All authors read and approved the final manuscript.

## Pre-publication history

The pre-publication history for this paper can be accessed here:

http://www.biomedcentral.com/1471-2407/10/669/prepub
